# A rapid, parasite-dependent cellular response to *Dirofilaria immitis* in the Mongolian jird (*Meriones unguiculatus*)

**DOI:** 10.1186/s13071-020-04455-x

**Published:** 2021-01-07

**Authors:** Christopher C. Evans, Katherine M. Day, Yi Chu, Bridget Garner, Kaori Sakamoto, Andrew R. Moorhead

**Affiliations:** 1 Department of Infectious Diseases, College of Veterinary Medicine, University of Georgia, Athens, GA 30602 USA; 2 Department of Pathology, College of Veterinary Medicine, University of Georgia, Athens, GA 30602 USA

**Keywords:** *Brugia malayi*, *Dirofilaria immitis*, Immunology, In vitro, Jird, Third-stage larvae

## Abstract

**Background:**

The Mongolian jird (*Meriones unguiculatus*) has long been recognized as a permissive host for the filarial parasite *Brugia malayi*; however, it is nonpermissive to another filarial parasite, canine heartworm (*Dirofilaria immitis*). By elucidating differences in the early response to infection, we sought to identify mechanisms involved in the species-specific clearance of these parasites. We hypothesized that the early clearance of *D. immitis* in intraperitoneal infection of the jird is immune mediated and parasite species dependent.

**Methods:**

Jird peritoneal exudate cells (PECs) were isolated and their attachment to parasite larvae assessed in vitro under various conditions: *D. immitis* and *B. malayi* cultured separately, co-culture of both parasites, incubation before addition of cells, culture of heat-killed parasites, and culture with PECs isolated from jirds with mature *B. malayi* infection. The cells attaching to larvae were identified by immunohistochemistry.

**Results:**

In vitro cell attachment to live *D. immitis* was high (mean = 99.6%) while much lower for *B. malayi* (mean = 5.56%). This species-specific attachment was also observed when both filarial species were co-cultured, with no significant change from controls (*U*_(9, 14)_ = 58.5, *p* = 0.999). When we replicated these experiments with PECs derived from jirds subcutaneously infected with *B. malayi*, the results were similar (99.4% and 4.72% of *D. immitis* and *B. malayi*, respectively, exhibited cell attachment). Heat-killing the parasites significantly reduced cell attachment to *D. immitis* (mean = 71.9%; *U*_(11, 14)_ = 7.5, *p* < 0.001) while increasing attachment to *B. malayi* (mean = 16.7%; *U*_(9, 15)_ = 20, *p* = 0.002). Cell attachment to both species was reduced when larvae were allowed a 24-h pre-incubation period prior to the addition of cells. The attaching cells were identified as macrophages by immunohistochemistry.

**Conclusions:**

These results suggest a strongly species-dependent response from which *B. malayi* could not confer protection by proxy in co-culture. The changes in cell attachment following heat-killing and pre-incubation suggest a role for excretory/secretory products in host immune evasion and/or antigenicity. The nature of this attachment is the subject of ongoing study and may provide insight into filarial host specificity.
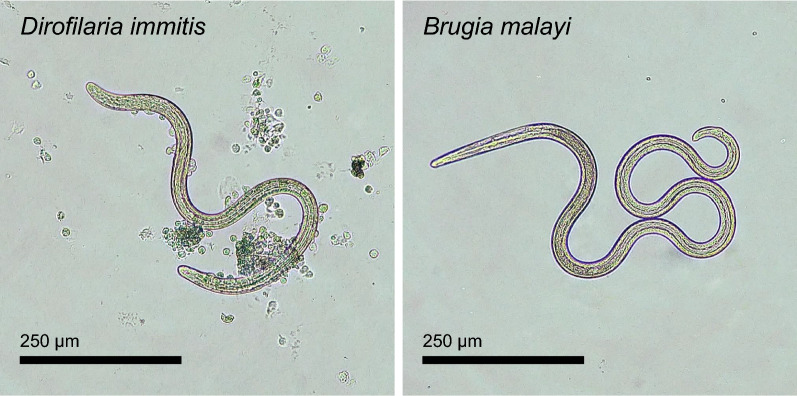

## Background

The Mongolian jird (*Meriones unguiculatus*) has long been recognized as a permissive host for the filarial nematode *Brugia malayi*, and this quality, combined with ease of handling and housing, has made the jird a preferred laboratory model for the study and propagation of this parasite [1–3]. These animals can be infected by multiple routes, with differing infection characteristics. The subcutaneous route of infection involves injection by needle of infective third-stage larvae (L3) and mimics the route of natural, mosquito-acquired infections; as a result, parasites migrate into their natural predilection sites in the lymphatics, where their recovery is challenging. The intraperitoneal route of infection, in which L3 are injected by needle into the peritoneal cavity, is particularly useful in that adult worms largely remain confined therein and can be easily recovered; this is an advantage of the jird over other rodents, such as *Mastomys* spp., which do not support intraperitoneal infection [4, 5], and wild-type mouse strains, which are not permissive to *B. malayi* infection by any route [6, 7]. However, this parasite has been demonstrated to reach maturity in certain immunodeficient mouse models [7–9]. It seems reasonable, therefore, to ascribe the permissivity of the jird in large part to its species-specific immunological characteristics.

The jird, however, is not considered to be permissive to canine heartworm (*Dirofilaria immitis*), which, like *B. malayi*, is a filarial nematode of the family Onchocercidae [10]. Preliminary experiments for the present study corroborated these findings, in which we observed poor recovery of *D. immitis* L3 inoculated via the intraperitoneal route (200 L3 per animal) after 1 h (19.5%, n = 3), 2 h (3%, *n* = 1) and 24 h (2%, *n* = 2). In contrast, *B. malayi* L3 routinely develop to reproductive adults in this system (29%, *n* = 30; unpublished data) with approximately 90% of L3 recoverable at 7 days post-infection (M. T. Dzimianski, personal communication). The strikingly different fates of these two, closely related filarial species invited a focused investigation into the nature of the host response.

In the natural canine host, *D. immitis* L3 penetrate the bite wound left by the mosquito vector and rapidly enter the subcutaneous tissues. In experimental infections, most larvae are recoverable in the vicinity of the inoculation site 3 to 7 days later, suggesting little initial migration [11, 12]. The molt from L3 to L4 occurs 2 to 5 days post-infection, with the final molt occurring 50 to 70 days post-infection [13–16]. Aberrant arrival of filarial larvae in the pulmonary alveoli is followed by their immune-mediated death, so *D. immitis* avoids the cardiopulmonary system until attaining a suitable size (20–40 mm in length) at day 70–85 post-infection [16–18]. In contrast, *B. malayi* L3 are most often recovered from the lymphatics as early as 3 days post-infection in the cat and from the lymphatics and peritoneal cavity 2 days post-infection in the jird [18, 19]. This suggests that infection of the vertebrate host is followed quickly by migration away from the inoculation site, before the molt to L4 (8–10 days post-infection) [18, 20].

Filarial parasites are already well recognized to modulate the host immune system in ways that allow for their continued tolerance and survival, with much evidence supporting the involvement of parasite excretory/secretory (E/S) products [21–23]. It remains possible that variations between filarial species in the protective or immunogenic properties of their E/S products may be a determinant of host specificity, accounting for the long-term survival of *B. malayi* in the jird, while *D. immitis* is quickly cleared. Alternatively, the composition of the infective stage larval cuticle may elicit an immune response that E/S products cannot moderate. The aim of the present study was to verify and elucidate differences in early infection of the jird with *B. malayi* and *D. immitis* utilizing both in vivo and in vitro approaches. We hypothesized that the early clearance of *D. immitis* in intraperitoneal infection of the jird is immune mediated and parasite species dependent. We tested this by isolating jird peritoneal exudate cells (PECs) and L3 of both filarial species followed by in vitro culture. Parasite species were cultured separately, co-cultured to assess the protective properties of *B. malayi* E/S products by proxy, and after heat-killing to assess the host cell response when E/S products were no longer actively released. PECs derived from jirds with mature *B. malayi* infections were also investigated. Finally, the identity of the cell type observed attaching to *D. immitis* L3 was determined by microscopic examination of stained specimens.

## Materials and methods

### Parasites

L3 of *B. malayi* (FR3 strain) and *D. immitis* (MO 2005 strain) were collected from mosquitoes fed 15 days prior on microfilaremic cat and dog blood, respectively. All the parasites were provided by the Filariasis Research Reagent Resource Center (FR3; University of Georgia, Athens, GA). Larvae were isolated in Hanks’ balanced salt solution supplemented with penicillin, streptomycin, and gentamicin according to standard procedures (http://www.filariasiscenter.org/protocols).

### In vitro culture

Adult male jirds were euthanized by CO_2_ asphyxiation followed by cervical dislocation, and PECs were collected by peritoneal lavage. Unless otherwise specified, all jirds were naïve to filarial infection. The peritoneal cavity of each animal was lavaged with approximately 20 ml RPMI-1640 via a 5-mm abdominal incision. After of the full volume of exudate was collected in a 50-ml conical centrifuge tube, fresh RPMI-1640 was added to a final volume of 50 ml and centrifuged for 10 min at 650 *g*. After removing supernatant to reduce the volume to approximately 2 ml, RPMI-1640 supplemented with penicillin, streptomycin, gentamicin, ciprofloxacin, and Fungin (InvivoGen, San Diego, CA) was added to dilute to a PEC concentration of 5 × 10^5^ cells/ml upon vortexing. The total number of PECs collected per animal ranged from approximately 5 × 10^5^ to 1 × 10^6^. A serum-free culture medium was utilized to minimize the variability in medium composition while still allowing survival to the point that the cell attachment phenomenon of interest may be observed.

L3 of *B. malayi* and *D. immitis* were transferred in a 200-µl volume by pipetting to a 24-well culture plate (20 L3 per well, six wells per species). This 24-well plate format, described previously [24, 25], allowed easy visualization of cell attachment to each larva. In those experiments where the two species were co-cultured, ten L3 of each species were transferred to each well for a total of 20 L3; this controls for nutrient consumption and well area occupancy. For experiments with a pre-incubation period, L3 were cultured alone for 24 h before PECs were collected and added to the culture plate; subsequent culture procedures were identical to those described above. For experiments requiring heat-killed larvae, parasites were heated in 1.5-ml microfuge tubes and placed in a heat block at 65 °C for 15 min before transfer to the culture plate. For culture conditions utilizing PECs from mature filarial infections, cells were collected from adult male jirds harboring infections with subcutaneously inoculated *B. malayi* (infected 118–120 weeks prior with 100 L3 per animal) as described above for naïve jirds; larvae were cultured as described above, separated by species.

In each experiment, half of all wells received 1 ml PEC suspension, while the other half received antibiotic-supplemented RPMI as a cell-free control to assess larval survival. Larva-cell co-cultures were incubated at 37 °C and 5% CO_2_ for 20 h. Following incubation, larvae were examined using an inverted compound microscope (Nikon TMS; Nikon) at 100× magnification. Larvae were assessed for cell attachment and survival. Attachment was defined as one or more PECs visibly attached to the cuticle of the nematode even when disturbed; because cell attachment to *B. malayi* was almost always absent, the presence of any attachment was considered noteworthy and a dichotomous scoring system was selected to allow for more robust statistical analysis. Survival was defined as at least one motility event within 6 s of observation. In those wells containing both *B. malayi* and *D. immitis*, cell attachment and survival were assessed by species; non-overlapping size differences between species at this developmental stage allowed reliable identification. All experiments were performed with three to five replicates.

### Cell identification

After 20 h of culture with PECs, *D. immitis* L3 with cell attachment were removed from the culture plate by pipetting and concentrated in a 1.5-ml centrifuge tube. Excess media was removed by pipetting, and specimen processing gel (HistoGel; Richard-Allan Scientific, San Diego, CA) heated to 60 °C was added to embed larvae. These samples were fixed in 10% neutral-buffered formalin for 20 h then submitted to the University of Georgia Histology Laboratory (Athens, GA) for routine processing, sectioning at 5-µm thickness, and staining with hematoxylin and eosin stains. Jird spleen sections were prepared in the same way as a positive control for immunoreaction in this species.

Immunohistochemistry was performed on an automated stainer (Nemesis 3600; Biocare Medical, Concord, CA). For the detection of T lymphocytes, a rabbit polyclonal antibody for cluster of differentiation 3 (CD3) (A045201; Agilent Technologies) was used at a dilution of 1:1000 for 60 min. Antigen retrieval on tissue sections was achieved using Citrate Plus Solution (10× Concentrated) (BioGenex, Fremont, CA) at a dilution of 1:10 for 15 min at 110 °C. A biotinylated goat anti-rabbit antibody at a dilution of 1:100 (Vector Laboratories, Burlingame, CA) was utilized to detect the target. For the detection of B lymphocytes, a mouse monoclonal antibody for CD79a was used at a dilution of 1:50 for 60 min. Antigen retrieval was achieved using Reveal (Biocare Medical) at a dilution of 1:10 for 15 min at 110 °C. A biotinylated horse anti-mouse antibody at a dilution of 1:100 (Vector Laboratories) was utilized to detect the target. For the detection of macrophages, a rabbit polyclonal antibody for ionized calcium-binding adaptor molecule 1 (IBA1) (019-19741; Wako Chemicals, Richmond, VA) was used at a dilution of 1:8000 for 60 min. Antigen retrieval on tissue sections was achieved using Citrate Plus Solution (10× Concentrated) (BioGenex) at a dilution of 1:10 for 15 min at 110 °C. A biotinylated goat anti-rabbit antibody at a dilution of 1:100 was utilized to detect the target. In all cases, immunoreaction was visualized using 3,3-diaminobenzidine (Biocare Medical) substrate for 12 min counterstained with hematoxylin. Lymph node tissue was used as a positive control for CD3 and CD79a reactions, while spinal cord tissue was used as a positive control for IBA1.

### Data analysis

Cell attachment data were not normally distributed. Therefore, a two-tailed Mann–Whitney* U*-test was performed for each comparison of cell attachment rates, applying Bonferroni correction. The survival rates of larvae between groups with and without jird PECs were similarly compared. Analyses were performed using GraphPad Prism version 6 (GraphPad Software, San Diego, CA).

## Results

### In vitro culture

**Fig. 1:**
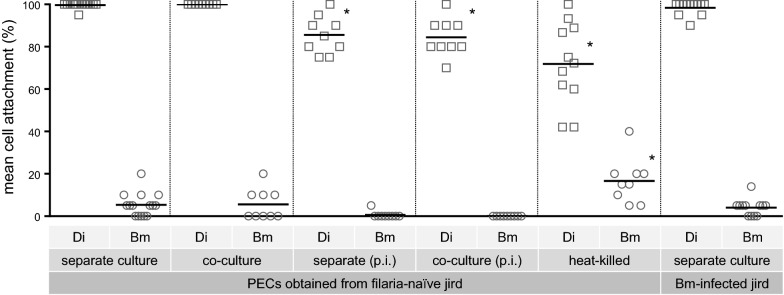
Mean percent third-stage larvae (L3) of *Dirofilaria immitis* (*Di*) and *Brugia malayi* (*Bm*) exhibiting jird peritoneal exudate cell (*PEC*) attachment is presented for each culture condition. Larvae were incubated separately by species (*separate culture*), together in equal numbers (*co-culture*), with a 24-h pre-incubation (*p.i.*) period before exposure to jird cells, or heat-killed. The source of jird cells is indicated beneath the condition tested, i.e., either filaria-naïve jird or jirds previously infected with *B. malayi* via the subcutaneous route. For all conditions, we cultured 20 larvae per well.* Asterisks* indicate a significant intraspecific difference from culture conditions in the first group (*p* < 0.05)

When cultured separately, almost all *D. immitis* L3 demonstrated attachment by one or more jird-derived PECs when examined at 20 h (mean = 99.6%), while *B. malayi* demonstrated very low levels of attachment (mean = 5.3%); this difference was significant (*U*_(14, 15)_ = 0, *p* < 0.001; Fig. 1). Similar results were observed when both species were co-cultured, with no significant change in cell attachment to *D. immitis* in the presence of *B. malayi* (*U*_(9, 14)_ = 58.5, *p* = 0.999). Culturing L3 for 24 h prior to the addition of PECs, thus allowing accumulation of larval E/S products, resulted in a decrease in cell attachment for both species, which was similar for both separate and co-cultures; this decrease in comparison to the culture group without pre-incubation was significant for *D. immitis* under both conditions (*U*_(9, 14)_ = 8, *p* < 0.001). Reducing E/S product release by heat-killing yielded significantly reduced cell attachment in *D. immitis* compared to the live control group (mean = 71.9%; *U*_(11, 14)_ = 7.5, *p* < 0.001), while cell attachment to *B. malayi* was significantly increased (mean = 16.7%; *U*_(9, 15)_ = 20, *p* = 0.002). Nonetheless, attachment rates between heat-killed worms of each species remained significantly different (*U*_(11, 9)_ = 0, *p* < 0.001). In culture conditions where PECs were derived from jirds bearing mature *B. malayi* infection, we observed similar results as with filaria-naïve jirds (mean = 99.4% and 4.72% of *D. immitis* and *B. malayi*, respectively), with no significant change in attachment to *D. immitis* (*U*_(12, 14)_ = 68.5, *p* = 0.255) or *B. malayi* (*U*_(12, 15)_ = 80, *p* = 0.621). A high degree of variability in the number of cells attaching to *D. immitis* L3 was noted, from a single cell to a number that covered approximately 90% of the length of the larva; the number of cells attaching to *B. malayi* L3, however, were always very few, covering no more than approximately 10% of the length of the worm.

Survival of *B. malayi* was high in the presence or absence of PECs (98.4% and 98.6% pooled among all groups, respectively), while *D. immitis* demonstrated a modest but statistically insignificant reduction in survival in the presence of PECs (89.2% versus 96.1% pooled among all groups; Additional file 1: Fig. S1).

### Cell identification

**Fig. 2:**
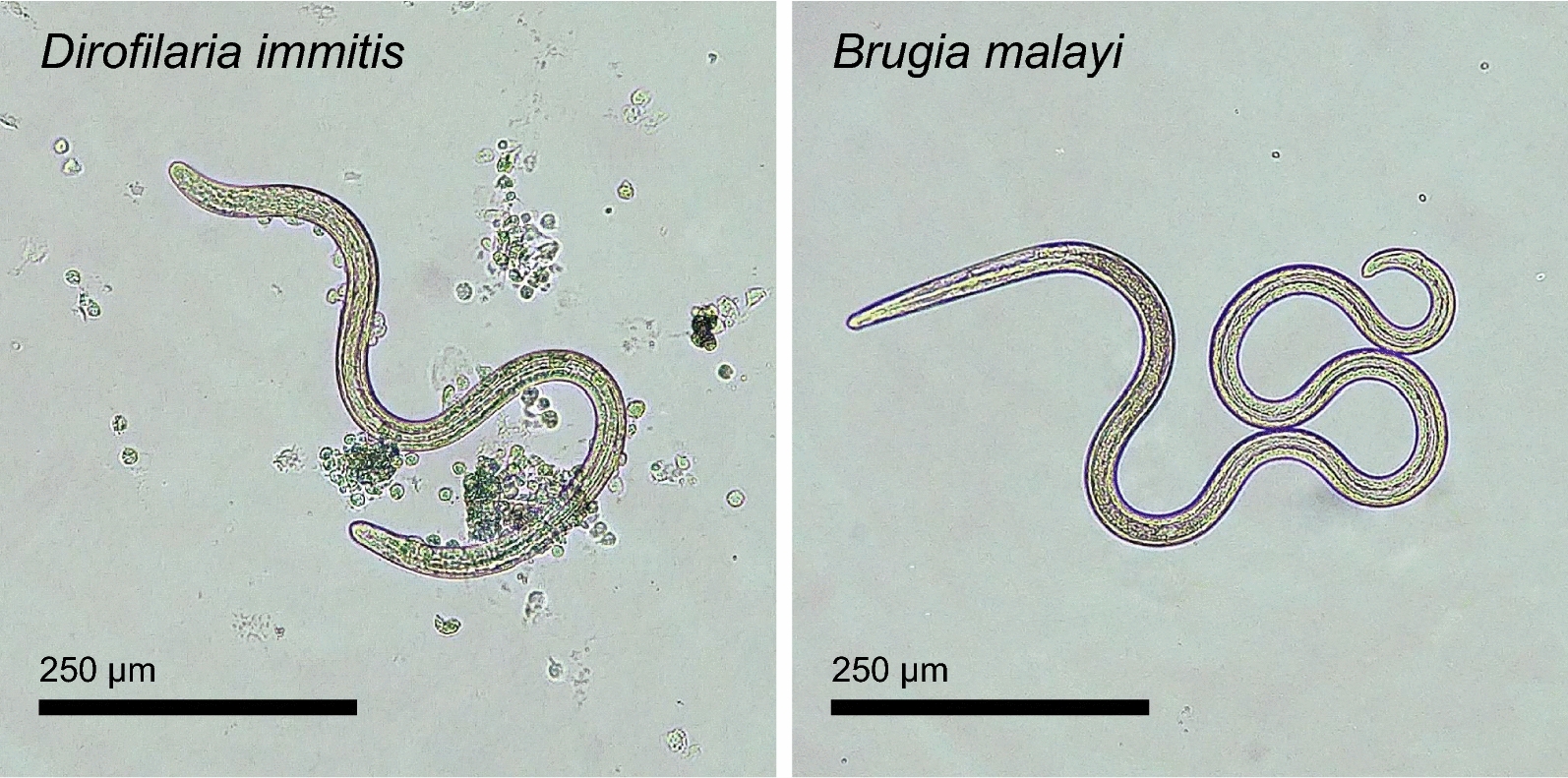
L3 of *D. immitis* and *B. malayi* following 20 h culture with jird PECs. Host cell attachment was typically observed on *D. immitis* but not on *B. malayi*

**Fig. 3:**
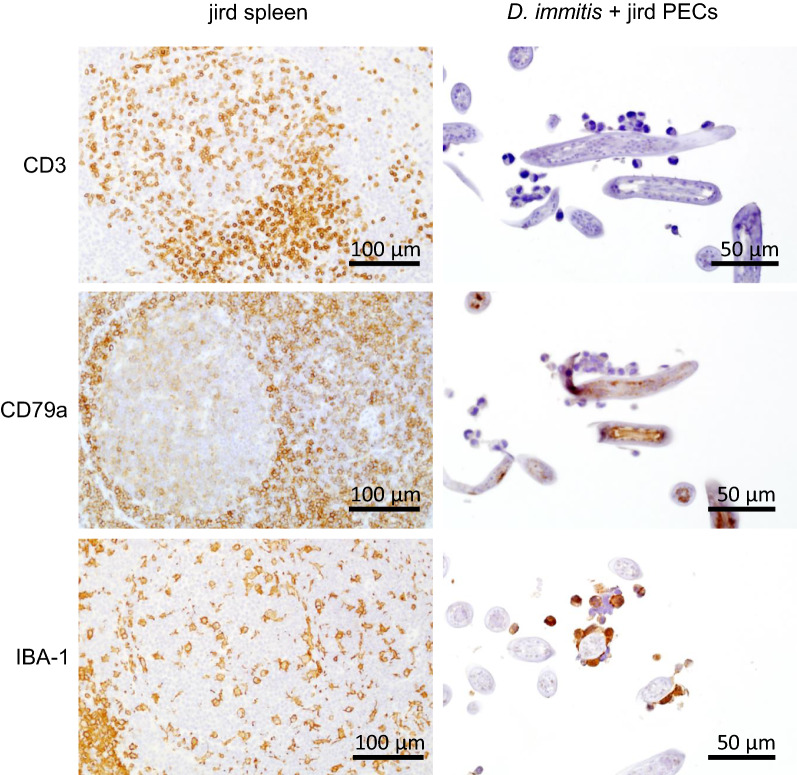
Immunohistochemistry of L3 of *D. immitis* with attached jird PECs following 20 h co-culture. Hematoxylin and eosin staining was followed by immunolabeling for cluster of differentiation 3 (*CD3*), CD79a, and ionized calcium-binding adaptor molecule 1 (*IBA1*). Jird spleen section served as a positive control for each

The subpopulation of PECs found associated with L3 were mononuclear and nongranular (Fig. 2). These cells demonstrated positive immunolabeling for IBA1 and a lack of immunolabeling for CD3 and CD79a, consistent with a macrophage phenotype (Fig. 3). Jird spleen sections served as a positive control for these cell types and demonstrated strong labeling in all reactions.

## Discussion

While *B. malayi* reliably develops to patency in the jird, previous attempts to establish *D. immitis* infections have been less successful [10]. We corroborated these findings in preliminary experiments, infecting via the intraperitoneal route and observing poor recovery rates at 1 h, 2 h and 24 h post-infection. Furthermore, the high rate of cellular attachment to recovered larvae suggested that a host response effected by innate mechanisms may be responsible for their rapid clearance and/or sequestration. Working from these findings, we focused on the subpopulation of cells present in the jird peritoneal cavity.

Through a series of in vitro co-culture experiments, we repeatedly observed high rates of jird PEC attachment to *D. immitis* L3 contrasted with very little attachment to *B. malayi* L3. This agreed with our in vivo preliminary findings, and suggested that the same cellular response was being observed. These results were not affected by the co-culture of both parasites or by the infection status of the donor jird (Fig. 1). The co-culture condition was examined to evaluate the protective properties of *B. malayi* E/S products on *D. immitis*, which may benefit from their release by proxy; our observations suggest that the immunomodulatory effect *B. malayi* may be generating through these mechanisms is not imparted to neighboring parasites. Furthermore, *D. immitis* was no less likely to exhibit attachment by PECs collected from jirds with established *B. malayi* infections than those from naïve jirds, suggesting that long-term immunomodulation is not protective against this cellular response. Indeed, resistance to reinfection in animals harboring adult filarial worms is a known phenomenon [26]. It should be noted, however, that these infections were not established via the intraperitoneal route, and so the PEC subpopulation may not be representative of immune cell populations at the parasite’s predilection site or along the natural route of migration. In future investigations, we will utilize PECs derived from intraperitoneally infected jirds.

In the present study, PEC attachment was significantly altered from baseline levels under only two conditions: pre-incubation of the parasite and utilization of heat-killed parasites. Under the former condition, L3 are expected to release E/S products into the surrounding culture media [22], conditioning their surroundings prior to the addition of any host cells. Median PEC attachment to both *D. immitis* and *B. malayi* was reduced compared to that of cultures without this pre-incubation period, with the differences in *D. immitis* being statistically significant (99.6–85.6% for separate culture and 100–84.4% for co-culture; *U*_(9, 14)_ = 8, *p* < 0.001). Jird PECs were collected just prior to their addition to culture, as in other conditions, suggesting that the differences seen here were due to a change in the parasite and/or its surroundings that reduced immunogenicity. In experiments with heat-killed L3, the neuromuscular control predicted to regulate the release of products at the E/S pore [27] is expected to be inhibited upon the death of the parasite, which provided us another means of assessing the relative influence of active excretion/secretion on host cell attachment. PEC attachment to *D. immitis* was significantly reduced (99.6–71.9%; *U*_(11, 14)_ = 7.5, *p* < 0.001), while attachment to *B. malayi* was significantly increased (5.3–16.7%; *U*_(9, 15)_ = 20, *p* = 0.002). While changes to cell attachment rates in both species are relatively modest, they suggest at least a partial effect of the active release of E/S products: immunogenic in the case of *D. immitis* and immunomodulatory in the case of *B. malayi*. This is in contrast to the reduction in cell attachment to 24-h pre-incubated *D. immitis* noted above. Previous reports demonstrate the release of E/S products by L3 of both of these species of filarial parasite and indicate their putative immunomodulatory effects [22, 23, 28, 29]. The secretome differences noted between these parasites [30] represent a possible determinant of host specificity and may be reflected in the ability of jird immune cells to recognize and respond to larvae. In the case of *D. immitis*, E/S products may either stimulate or modulate the rapid immune response we observed, depending on the time allowed for their production and accumulation.

Despite the effects of pre-incubation and heat-killing, PEC attachment rates remained significantly different between *D. immitis* and *B. malayi*. This species-specific response may be explained by constitutive differences in the L3 cuticle, an integral component of the host-parasite interface. In addition to their presence in solution, E/S products are observed to occur on the parasite surface and, thus, may aid in immune evasion [31]. The persistence of these products on the parasite cuticle, even after heat-killing, may explain the limited deviations from results obtained in live culture.

The cuticle is a complex structure secreted by the underlying hypodermal cell layer and changes composition over the course of a parasite’s life cycle. In the larval stages, during which the esophageal opening is completely occluded, it is a critical route of nutrient uptake. Each stage of the filarial parasite produces characteristic cuticular collagens not shared with other stages, and collagen synthesis increases during the process of molting [32]. While these collagens are located within the matrix of the cuticle where they are inaccessible to antibodies and other antigen receptors, they become exposed to the host environment after molting, and thus may serve as immunogenic factors [32, 33]. The timing of the molt from L3 to L4 differs between *D. immitis* and *B. malayi*: 2–3 days and 8–10 days post-infection, respectively [11, 15, 20]. It is not unlikely, therefore, that early molting events are underway in *D. immitis* larvae within the first day of isolation from the mosquito host. Furthermore, the requirements for molting to L4 in an in vitro system differ between species; ascorbic acid is demonstrated to aid molting in *B. malayi*, for instance, but is not required for *D. immitis* [34–36]. Media composition also influences the progression of the molt (e.g., serum-free media, for example, may allow development of the L4 epicuticle, but not ecdysis) [36]. The differences in molting status between these species may have differed over the course of the in vitro assays conducted in this study, and the impact this may have had on the observed cellular response cannot be discounted.

Filarial larvae are also known to present cuticular surface-associated proteins, some of which share identities with E/S products released during in vitro culture [31]. Several such proteins are expressed abundantly in the infective L3 (encoded by abundant larval transcripts or *alt*s) and are thus implicated in host-invasion events. In *B. malayi*, ALT1 and ALT2 are small secreted proteins expressed only at the larval stages; the homolog in *D. immitis* (Di20/22L) is secreted by the L3 and L4 stages around the time of the molt [29, 37]. These proteins share 57.8% amino acid homology, and differences between these and other secreted proteins may influence the host reaction [37]. The L3 of *D. immitis* actively shed surface products (a 35-kDa polypeptide and 6- to 10-kDa glycolipid) early in development both in vitro and in vivo, which appears to reduce their antigenicity and help them to evade early host immune responses [38]. A surface antigen recognized by a monoclonal antibody against *B. malayi* is also rapidly shed from the parasite upon removal from the vector and incubation at 37 °C [39]. The most prominent noncollagenous component of the filarial cuticle, however, is a 29-kDa glycoprotein (gp29, or *B. malayi* GPX1) with functions that may provide protection against oxidative immune responses, such as those effected by macrophages and neutrophils; this protein is present in the L3 of *Brugia pahangi*, but was first seen in the L4 of *B. malayi* and *D. immitis* [40–42]. The parasite cuticle may also adsorb proteins during development in the vector, and as a result of differing migratory routes collect different sets of antigenic substances even in the same mosquito species. The larvae of *D. immitis* develop primarily in the Malpighian tubules of the mosquito, while *Brugia* spp. localize in the flight muscles [43, 44]. By virtue of their developmental predilection sites, differences in decoration of the cuticular surface may thus affect how each species is recognized by the host upon infection. The variations in cuticular composition between species may reflect the various mechanisms relevant to their survival within their respective hosts, and as such, the immunogenic and/or immunomodulatory properties of the cuticle of each species warrant further investigation into their role in the early cellular response observed in the present study.

Furthermore, the migration strategies of the two species differ greatly in the hours to days following natural infection of the vertebrate hosts, in particular the timing of migration away from the site of inoculation; infective larvae of *B. malayi* and *B. pahangi* exhibit a rapid migration toward lymphatic vessels [19, 45], while *D. immitis* is recoverable in the subcutaneous tissue near the site of infection multiple days later [11, 12]. It would be unsurprising, therefore, if these parasites employ different means of immune evasion early in infection, and that these differences may be reflected in our in vitro results.

The peritoneal cavity of the jird is populated by several types of immune cells that parasites injected therein may encounter. Previous work by our group has identified neutrophils (i.e., heterophils), eosinophils, macrophages, and occasional lymphocytes and mast cells/basophils in peritoneal lavage fluid utilizing Wright-Giemsa staining of cytocentrifuge preparations (unpublished data). The rapid cellular response we observed against *D. immitis* appears to be effected by peritoneal macrophages, as determined by immunohistochemistry. The role of macrophages in anti-filarial immunity appears to be significant, as a recently developed rodent model for *D. immitis* successfully utilized immunodeficient NSG (NOD *scid* gamma) and NRG (NOD* rag* gamma) mice to mature this parasite to the adult stage [46]; both NSG and NRG mice possess defective macrophages and dendritic cells and are deficient in lymphocytes, innate lymphoid cells, and natural killer (NK) cells; thus, macrophages alone or complemented by another of these cell types may be responsible for the clearance of *D. immitis* observed in immunocompetent wild-type mice. Subcutaneous infection of mice with *B. malayi* induces an early spike in IL-4 in the draining lymph nodes, attributed to NKT cells, which induces alternatively activated macrophages [47, 48]. Intraperitoneal infection with *B. malayi* also induces this polarization of macrophages [49]. This subpopulation of macrophages is activated by type 2 cytokines and downregulates potentially pathogenic inflammatory Th1-type reactions. They are observed in a broad variety of helminth infections [50]. In murine infections with *B. malayi* L3 (both subcutaneous and intraperitoneal), alternatively activated macrophages exhibit IL-10-independent antiproliferative effects on lymphocytes [51, 52].

Macrophages are also the primary cell type involved in the formation of granulomas, which sometimes surround adult filarial worms in the jird peritoneal cavity [53]. The involvement of macrophages in parasite establishment is further demonstrated by studies showing an abatement of age- and sex-related differences in permissivity of mice to *B. pahangi* when macrophage activity is inhibited [54].

Lymphocytes of the B1 subclass are activated by T-independent antigens, respond rapidly to infection, and occur predominantly in the peritoneal cavity, suggesting possible involvement in other hosts. Jirds, however, conspicuously lack this cell type, and its absence has been implicated in these rodents’ susceptibility to *B. malayi* infection [8, 55]; it should be noted, however, that the B1-deficient mouse strain (*xid*) utilized to demonstrate the importance of that cell type also exhibits altered macrophage activity [56]. Type 2 innate lymphoid cells (ILC2s) are early responders to helminth infection, secreting cytokines that promote protective immunity [57], and may be responsible for the initial rapid response to *D. immitis* in the jird, if not its clearance. ILC2 are implicated in the protection against other filarial infections in rodent models, along with NK cells, another subset of innate lymphoid cell [58]. Contact with *B. malayi* has been shown to induce both type 1 and type 2 cytokine secretion by NK cells in vitro, which may be critical in driving the course of the host immune reaction [59]. The role of complement in this rapid recognition of parasites may also be relevant. The cuticles of *B. malayi* L3 bear complement fraction 3 (C3) conversion products when cultured with human serum [60], and when cultured with rat serum, larvae are killed in a complement-mediated manner [61]. Adults of *D. immitis* also bear C3 on the cuticular surface, potentially masking recognizable parasite antigens [62]. Furthermore, the NSG and NRG mouse strains permissive to *D. immitis* lack functional complement pathways.

## Conclusions

The species-specific permissivity of the jird to one filarial nematode and its nonpermissivity to another provides a unique opportunity for investigating the underlying mechanisms of parasite establishment. The differences in the response to L3 of *D. immitis* and *B. malayi* are clear and consistent, remaining intact even under in vitro conditions. Identifying the host pathway for this response against *D. immitis*, and the parasite factors involved in eliciting this response warrant further study. Extrapolating from the permissivity of other model organisms, including immunodeficient mice, may aid this work. Understanding the mechanisms of early *D. immitis* clearance can help us to exploit these immune factors and improve our knowledge of filarial host specificity.

## Supplementary information

**Additional file 1: Fig. S1.** Mean percent survival of *Dirofilaria immitis* (*Di*) and *Brugia malayi* (*Bm*) third-stage larvae (*L3*) is presented for each culture condition with (*plus sign*) and without (*minus sign*) the addition of jird peritoneal exudate cells (*PECs*). Larvae were incubated separately by species (*separate culture*), together in equal numbers (*co-culture*), or with a 24-h pre-incubation (*p.i.*) period before exposure to jird cells. The source of jird cells is indicated beneath the condition tested. For all conditions, we cultured 20 larvae per well

## Data Availability

The datasets supporting the conclusion of this article are included within the article and its additional files. Raw data are available from the corresponding author upon request.
