# Supplementary figures and images for: A rapid, parasite-dependent cellular response to Dirofilaria immitis in the Mongolian jird (Meriones unguiculatus)
Source: Parasit Vectors. 2021 Jan 7;14:25. doi: 10.1186/s13071-020-04455-x (PMC7788973; doi:10.1186/s13071-020-04455-x)

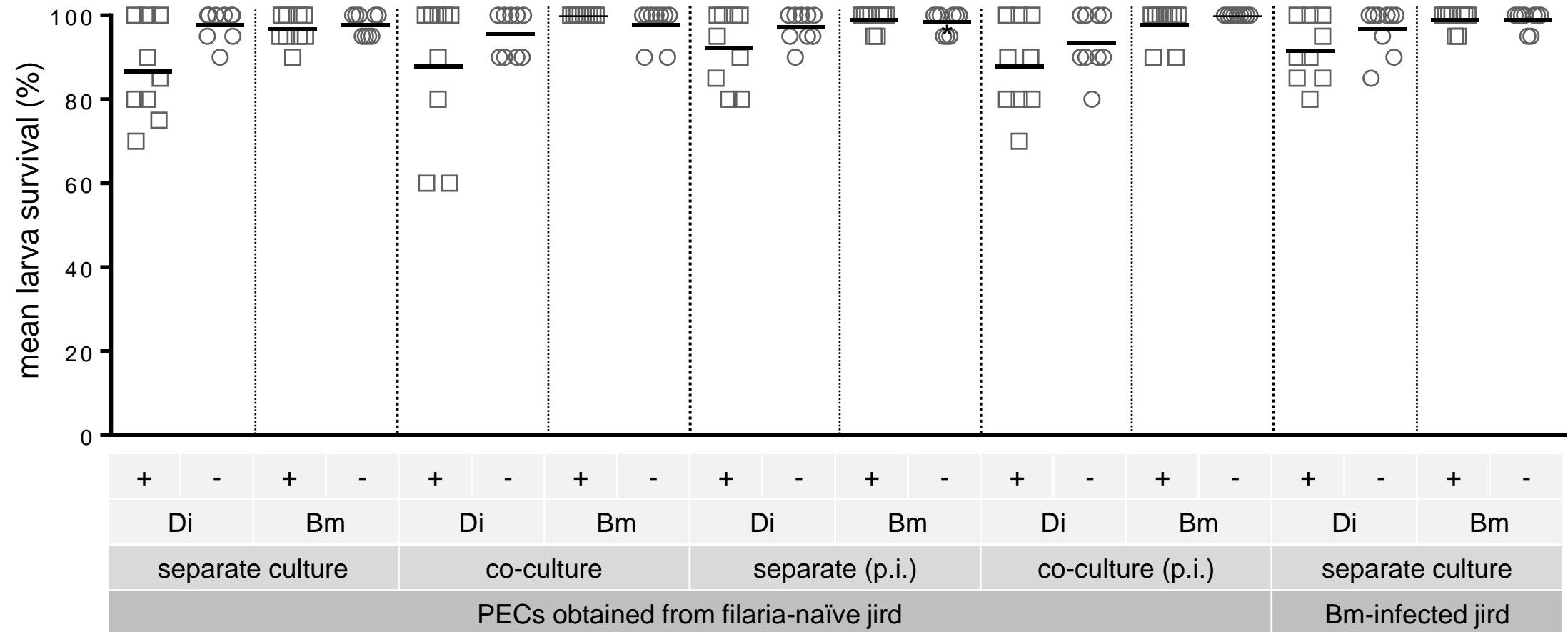

Supplement: Supplementary file 1 — Additional file 1: Fig. S1. Mean percent survival of Dirofilaria immitis (Di) and Brugia malayi (Bm) third-stage larvae (L3) is presented for each culture condition with (plus sign) and without (minus sign) the addition of jird peritoneal exudate cells (PECs). Larvae were incubated separately by species (separate culture), together in equal numbers (co-culture), or with a 24-h pre-incubation (p.i.) period before exposure to jird cells. The source of jird cells is indicated beneath the condition tested. For all conditions, we cultured 20 larvae per well [file 13071_2020_4455_MOESM1_ESM.pdf]
